# The relation between air pollution and respiratory deaths in Tehran, Iran- using generalized additive models

**DOI:** 10.1186/s12890-018-0613-9

**Published:** 2018-03-20

**Authors:** Azizallah Dehghan, Narges Khanjani, Abbas Bahrampour, Gholamreza Goudarzi, Masoud Yunesian

**Affiliations:** 1 Neurology Research Center, Kerman University of Medical Sciences, Kerman, Iran; 2 Environmental Health Engineering Research Center, Kerman University of Medical Sciences, Kerman, Iran; 3 Monash Centre for Occupational & Environmental Health, School of Public Health and Preventive Medicine, Monash University, Melbourne, Australia; 4 Physiology Research Center, Kerman University of Medical Sciences, Kerman, Iran; 5 Air Pollution and Respiratory Diseases Research Center, Ahvaz Jundishapur University of Medical Sciences, Ahvaz, Iran; 6 Environmental Technologies Research Center (ETRC), Ahvaz Jundishapur University of Medical Sciences, Ahvaz, Iran; 7 Department of Environmental Health Engineering, School of Public Health, Tehran University of Medical Sciences, Tehran, Iran; 8 Center for Air Pollution Research and Department of Research Methodology and Data Analysis, Institute for Environmental Research (IER), Tehran University of Medical Sciences, Tehran, Iran; 9 Department of Epidemiology and Biostatistics, School of Public Health, Kerman University of Medical Sciences, Kerman, Iran

**Keywords:** Air pollution, Death, Respiratory disease

## Abstract

**Background:**

Some epidemiological evidence has shown a relation between ambient air pollution and adverse health outcomes. The aim of this study was to investigate the effect of air pollution on mortality from respiratory diseases in Tehran, Iran.

**Methods:**

In this ecological study, air pollution data was inquired from the Tehran Province Environmental Protection Agency and the Tehran Air Quality Control Company. Meteorological data was collected from the Tehran Meteorology Organization and mortality data from the Tehran Cemetery Mortality Registration. Generalized Additive Models (GAM) was used for data analysis with different lags, up to 15 days. A 10-unit increase in all pollutants except CO (1-unit) was used to compute the Relative Risk of deaths.

**Results:**

During 2005 until 2014, 37,967 respiratory deaths occurred in Tehran in which 21,913 (57.7%) were male. The strongest relationship between NO_2_ and PM_10_and respiratory death was seen on the same day (lag 0), and was respectively (RR = 1.04, 95% CI: 1.02–1.07) and (RR = 1.03, 95% CI: 1.02–1.04). O_3_ and PM_2.5_ had the strongest relationship with respiratory deaths on lag 2 and 1 respectively, and the RR was equal to 1.03, 95% CI: 1.01–1.05 and 1.06, 95% CI: 1.02–1.10 respectively. NO_2_, O_3_, PM_10_ and PM_2.5_ also showed significant relations with respiratory deaths in the older age groups.

**Conclusions:**

The findings of this study showed that O_3_, NO_2_, PM_10_ and PM_2.5_ air pollutants were related to respiratory deaths in Tehran. Reducing ambient air pollution can save lives in Tehran.

## Background

Outdoor or indoor air pollution is caused by chemical, physical, and biological agents that change the normal composition of atmosphere. Household heating services, motor vehicles, industrial equipment, and forest fires are the most common sources of air pollution [1]. Nowadays air pollution is a complicated environmental health problem especially in developing countries [2]. Epidemiological evidence has shown relations between outdoor air pollution and various undesirable health consequences [2–5]. There are different ambient air pollutants which affect human health in different ways. The most important pollutants are particulate matter (PM), sulfur dioxide (SO_2_), nitrogen oxides (NO_x_), ozone (O_3_), and carbon monoxide (CO) [6].

Studies from Europe and America have showed that even short-term changes in air pollution can increase daily mortality [4, 5]. Although the relative risk of mortality caused by air pollution is low, the proportion of deaths related to air pollution is high because of the high number of at-risk and sensitive populations [7].

It is estimated that outdoor air pollution in urban and rural areas caused 3 million early deaths around the world in 2012 and 88% of these deaths occur in low and middle-income countries, mostly in south East Asia and the western Pacific. According to the WHO estimations, 14% of early respiratory deaths and 14% of lung cancers are probably caused by air pollution [8].

Several studies have been done around the world about air pollutants and human deaths. However, most of them were done in developed countries and few studies were conducted in the Eastern Mediterranean region [9].

Air pollution is one of the most important environmental health problems in Tehran, the capital of Iran which has a population of over 12 million people. Controlling air pollution has been complicated in this city and investigation about the different aspects of air pollution and its health effects is still necessary [10, 11]. In this paper, the impact of air pollutants on respiratory disease mortality in Tehran has been evaluated.

## Methods

### Study design and setting

This ecological and population based study was conducted to determine the relation between air pollution and respiratory deaths in Tehran, Iran in 2005–2014. Tehran is the largest city and capital of Iran with a population of over 12 million people.

### Air pollution, meteorological and death data

Concentrations of ambient air pollutants were collected from the Tehran Air Quality Control Company and the Tehran Province Environment Protection Agency. There are 36 air pollution monitoring stations in Tehran, of which 21 operate under the Tehran Air Quality Control Company and the others are managed by the Tehran Province Environment Protection Agency. Tehran has 22 municipality districts and there are one or more air pollution monitoring stations in each district. Only 7 stations had data for 10 years under study. Other stations were between 1 to 5 years old. For this reason, we used the average daily pollutants in these seven stations as an indicator of air pollution in the whole city. These stations record ambient air pollutant concentrations per hour. We used average daily values in the analysis. We estimated missing data by the Expectation- Maximization algorithm (EM).

Meteorological data including temperature and relative humidity which are possible confounding parameters were collected from the Tehran Meteorological Organization. We used the average of daily temperature and relative humidity in this analysis. We had no missing meteorological data.

Data about number of mortalities caused by respiratory diseases in days were collected from the Tehran cemetery (Behesht-e-Zahra) and according to the international classification of diseases (ICD10) with J00-J99 codes (all respiratory deaths). We used aggregated death data for the whole city.

### Statistical analysis

Descriptive statistics including mean, standard deviation, the 25th, 50th (median), 75th and 98th percentiles, minimum and maximum were computed by SPSS 16.

Then the multi-pollutant Generalized Additive Model (GAM) was used for estimating the Rate Ratio (RR) of air pollutants’ effect on respiratory mortality. This model is an expanded form of the Generalized Linear Model (GLM) and has high flexibility. This model has been used in many studies because it can adjust for non-linear confounding parameters such as seasonal changes, trends and the weather variables [12, 13].

$$ {\mathrm{Y}}_{\mathrm{t}}\sim \mathrm{Poisson}\ \left({\upmu}_{\mathrm{t}}\right) $$

$$ \mathrm{Log}\ {\upmu}_{\mathrm{t}}=\upalpha +\sum {\upbeta}_{\mathrm{i}}\left({\mathrm{X}}_{\mathrm{i}}\right)+\sum {\mathrm{S}}_{\mathrm{j}}\left({\mathrm{X}}_{\mathrm{j}}\right)+ season+ day\  of\ week $$

where Y_t_ denotes the daily number of deaths due to respiratory disease, βi is the coefficients for air pollutants (Xi) and denotes the log Relative Rate of death related with each 1 unit increase in CO and each 10 unit increase in all other pollutants; and Sj (Xj) are the smoothing functions of meteorological variables (temperature and relative humidity) [14]. The degree of freedom (df) for the smoothers is determined with Generalized Cross Validation (GCV) by using the mgcv package in R software.

In this study multi-pollutant models were used and pollutants with less than 0.2 correlation with the main predicting pollutant were entered into the model. All of the pollutants did not come into the model together, due to the high correlation between some pollutants.

In this part of the study, meteorology variables (humidity, temperature), season and day of the week were added to the model as potential confounders and their effects were adjusted by using smooth functions to determine the exact effects of pollutants on number of deaths. The time unit used in the analysis was day.

Using the formulas below, we computed the Relative Risk (RR) and 95% Confidence Interval (CI) for RR.

$$ \mathrm{RR}=\exp\ \left(\upbeta \right) $$

$$ 95\%\mathrm{CI}=\exp\ \left(\upbeta \pm 1.96\ \mathrm{SE}\right) $$

Microsoft Office Excel 2007 and SPSS 16 software were used to prepare the data, and the ‘mgcv’ package in R i386 3.2.2 software was used for GAM analysis.

## Results

In the 10-year period under study, 37,967 Respiratory deaths occurred in Tehran, in which 21,913 (57.73%) cases were male and 16,047(42.27) were female. The male to female sex ratio was 1.36, 9065 (23.87%) deaths occurred in spring, 8528 (22.46%) in summer, 9323 (24.55%) in autumn and 11,051 (29.1%) in winter.

The frequency of male, female and total respiratory deaths in 2005–2014 are shown in Table 1.

**Table 1:** Frequency of male, female and total respiratory deaths in 2005–2014

	2005	2006	2007	2008	2009	2010	2011	2012	2013	2014	Total
Male	1772	1731	1774	1873	1850	2257	2323	2511	2936	2886	21,913
Female	1272	1290	1302	1210	1415	1700	1763	1803	2126	2166	16,047
Sex Ratio	1.39	1.34	1.36	1.54	1.30	1.32	1.31	1.39	1.38	1.33	1.365
Age- standardized rate^a^	117.49	114.09	117.05	112.78	119.91	120.62	118.99	113.73	119.6	119.2	–

^a^Deaths per 100,000 population

In this study 13.1% of air pollution data was missing. The missing data was 10.51% in O3, 16.37% in CO, 8.73% in NO_2_, 10.51% in SO_2_, 7.09% in PM_10_ and 35.10% in PM_2.5_. They were estimated using the EM algorithm method.

The descriptive statistics of air pollution data, meteorological data and respiratory deaths are shown in Table 2.

**Table 2:** Descriptive statistics of daily air pollution, meteorological data and mortality

	Mean	SD	Min	25th Percentile	Median	75th percentile	98th percentile	Max
O_3_ (ppb)	24.66	9.125	6.89	18.31	23.30	28.98	48.70	77.09
CO (ppm)	3.29	0.97	1.47	2.61	3.13	3.81	5.81	11.29
NO_2_ (ppb)	53.47	16.52	12.06	43.03	49.82	60.33	97.04	153.57
SO_2_ (ppb)	33.46	10.47	9.02	26.87	32.05	38.26	62.08	119.10
PM_10_ (μg/m^3^)	69.75	20.61	12.02	57.39	68.04	80.65	117.2	331.20
PM_2.5_ (μg/m^3^)	20.68	5.87	7.43	16.72	20.23	23.67	35.85	55.06
Temperature (°C)	16.49	9.52	−8.70	8.30	16.80	25.20	31.60	34.40
Relative humidity (%)	43.72	18.37	9.75	29.63	39.50	55.75	88.62	89
Male deaths(N)	6.00	2.44	0	4	6	8	12	16
Female deaths(N)	4.39	1.90	0	3	4	6	9	16
Total deaths(N)	10.40	3.97	2	7	10	13	19	31

The result of this study showed that the daily average concentrations for PM_2.5_ was 20.68 μg/m3 and for PM_10_ was 69.75 μg/m3 and these two concentrations were higher than the WHO 2014 guideline annual thresholds (20 and 10 μg/m^3^, respectively). However, the 98th percentile of CO, O_3_ and SO_2_ was less than the WHO threshold.

In 3173 from 3652 days, the PM_10_ concentration was higher than the WHO guideline daily threshold (50 μg/m^3^). Also in 315 days from 1826 days, the PM_2.5_ concentrations were higher than WHO guideline daily threshold (25 μg/m^3^).

**Fig. 1:**
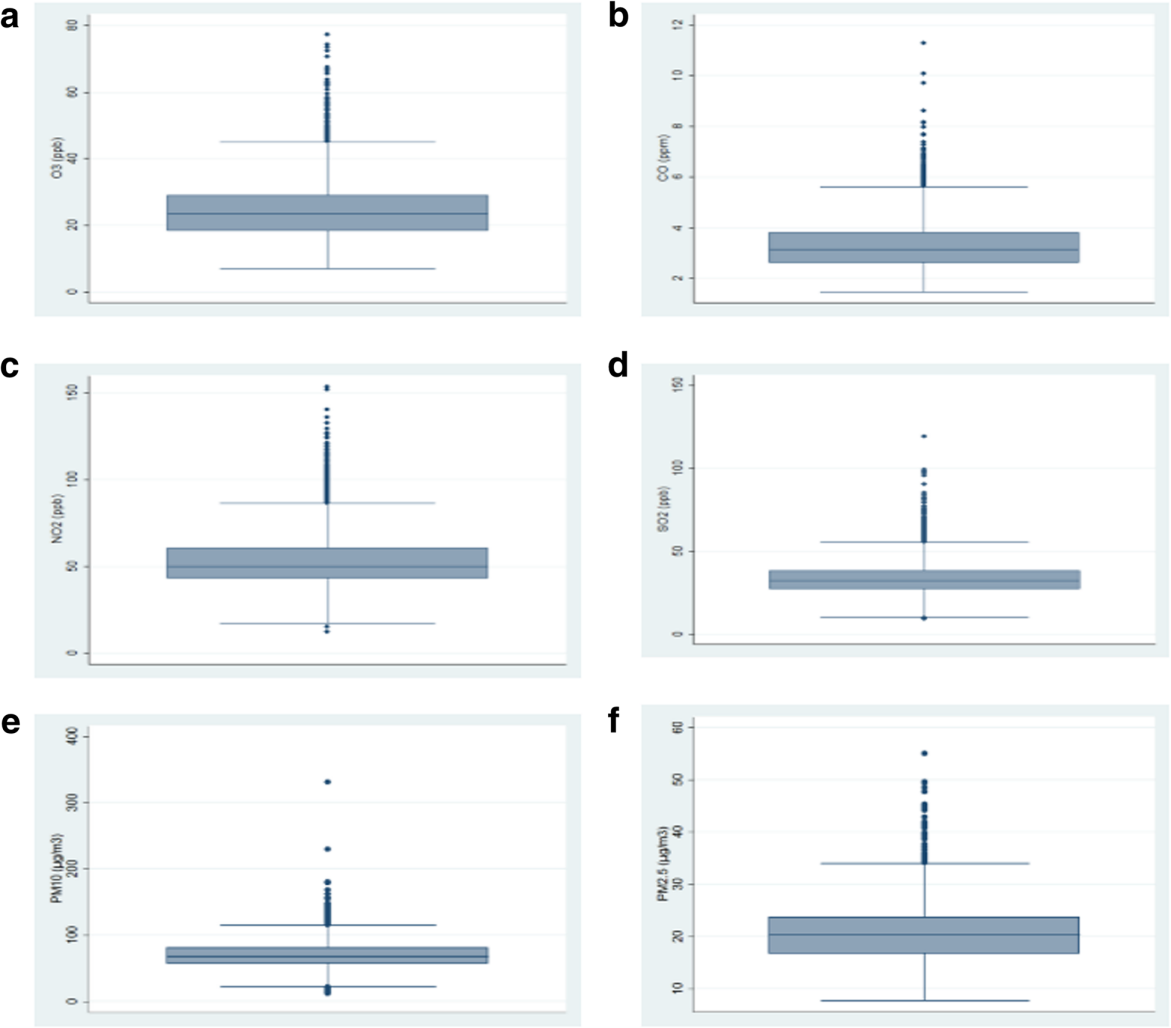
The box plots for air pollutants (**a**: O_3_, **b**: CO, **c**: NO_2_, **d**: SO_2_, **e**: PM_10_ and **f**: PM_2.5_)

The annual average of NO_2_ in 2004 to 2009 was higher, but in 2010 to 2013 was lower than WHO guidelines. The 75 percentile in all years was higher than WHO guidelines (50 ppb). Figure 1 shows the boxplots for all pollutants.

Table 3 shows the results of the adjusted Generalized Additive Model (multi-pollutant), about the effect of air pollutants on overall respiratory deaths. The table shows that ambient O_3_, and NO_2,_ PM_10_ and PM_2.5_ may affect respiratory death. The strongest relationships for each of these pollutants were in lag 2 for O_3_ (RR = 1.03, 95% CI; 1.01–1.05), lag 0 for NO_2_ (RR = 1.04, 95% CI; 1.02–1.07), lag 0 for PM_10_ (RR = 1.03, 95% CI; 1.02–1.04) and lag 1 for PM_2.5_ (RR = 1.06, 95% CI; 1.02–1.10).

**Table 3:** Results of adjusted Generalized Additive Model, about the effect of air pollutants on total respiratory deaths (for 1 unit increase in CO and 10 units increase in all other pollutants)

	Lag	df	RR	95% CI for RR	*P*-Value
Lag 0	O_3_ (ppb)	7.94	1.02	0.99–1.05	0.053
	CO (ppm)	5.04	1.02	0.99–1.05	0.139
	NO_2_ (ppb)	3.04	1.04	1.02–1.07	< 0.001
	SO_2_ (ppb)	2.61	0.96	0.93–0.98	0.006
	PM_10_ (μg/m^3^)	7.3	1.03	1.02–1.04	< 0.001
	PM_2.5_ (μg/m^3^)	3.26	1.04	1.00–1.08	0.008
Lag 1	O_3_ (ppb)	4.04	1.03	1.00–1.05	0.042
	CO (ppm)	6.35	0.96	0.99–1.05	0.130
	NO_2_ (ppb)	6.51	1.04	1.01–1.06	0.003
	SO_2_ (ppb)	3.65	0.95	0.92–0.98	0.004
	PM_10_ (μg/m^3^)	6.66	1.02	1.01–1.03	< 0.001
	PM_2.5_ (μg/m^3^)	2.69	1.06	1.02–1.10	< 0.001
Lag 2	O_3_ (ppb)	5.34	1.03	1.01–1.06	0.015
	CO (ppm)	5.79	1.03	0.99–1.06	0.199
	NO_2_ (ppb)	4.17	1.04	1.01–1.06	0.001
	SO_2_ (ppb)	4.21	0.95	0.92–0.98	0.004
	PM_10_ (μg/m^3^)	6.79	1.02	1.01–1.03	< 0.001
	PM_2.5_ (μg/m^3^)	2.41	1.04	1.00–1.08	0.001
Lag 3	O_3_ (ppb)	4.86	1.03	1.01–1.06	0.015
	CO (ppm)	5.08	1.02	0.99–1.05	0.228
	NO_2_ (ppb)	3.04	1.04	1.01–1.06	0.001
	SO_2_ (ppb)	8.14	0.95	0.92–0.98	0.004
	PM_10_ (μg/m^3^)	6.44	1.02	1.01–1.03	0.001
	PM_2.5_ (μg/m^3^)	2.56	1.03	0.98–1.07	0.064
Mean Lag 4–8	O_3_ (ppb)	3.42	1.02	0.99–1.05	0.103
	CO (ppm)	6.51	1.02	0.98–1.04	0.176
	NO_2_ (ppb)	5.79	1.03	1.01–1.06	0.004
	SO_2_ (ppb)	4.83	0.95	0.93–0.99	0.014
	PM_10_ (μg/m^3^)	6.94	1.01	1.00–1.02	0.002
	PM_2.5_ (μg/m^3^)	3.08	1.01	0.97–1.05	0.193

Tables 4 and 5 shows the effect of air pollutants on male and female respiratory deaths. Table 4 shows that O_3_, NO_2_, PM_10_ and PM_2.5_ had a direct association with respiratory deaths in males and females. In men, the strongest relation was seen in lag 0 for O_3_ (RR = 1.03, 95% CI; 1.00–1.06), in lag 0 for PM_10_ (RR = 1.03, 95% CI; 1.02–1.05), in lag 1 for NO_2_ (RR = 1.04, 95% CI; 1.00–1.06), and in lag 1 for PM_2.5_ (RR = 1.04, 95% CI; 1.02–1.07). In women, only NO_2_, PM_10_ and PM_2.5_ were related with respiratory death. NO_2_ and PM_10_ had the highest relation with respiratory deaths at lag 0 and PM_2.5_ at lag 1 in women. The Relative Risk of respiratory deaths for NO_2_, PM_10_ and PM_2.5_ was (RR = 1.06, 95% CI; 1.02–1.10), (RR = 1.03, 95% CI; 1.02–1.04) and (RR = 1.05, 95% CI; 1.01–1.09) respectively.

**Table 4:** Results of adjusted Generalized Additive Model and the effect of pollutants on respiratory deaths in men (for 1 unit increase in CO and 10 units increase in all other pollutants)

	Lag	df	RR	95% CI for RR	*P*-Value
Lag 0	O_3_ (ppb)	7.19	1.03	1.00–1.07	0.037
	CO (ppm)	4.88	0.98	0.94–1.01	0.235
	NO_2_ (ppb)	2.33	1.03	1.00–1.06	0.045
	SO_2_ (ppb)	1	0.96	0.92–1.01	0.093
	PM_10_ (μg/m^3^)	7.50	1.03	1.02–1.05	< 0.001
	PM_2.5_ (μg/m^3^)	3.92	1.02	0.97–1.08	0.156
Lag 1	O_3_ (ppb)	3.20	1.03	0.99–1.06	0.100
	CO (ppm)	6.45	0.97	0.94–1.01	0.107
	NO_2_ (ppb)	4.47	1.04	1.00–1.06	0.019
	SO_2_ (ppb)	3.15	0.95	0.91–0.99	0.032
	PM_10_ (μg/m^3^)	6.69	1.02	1.01–1.04	< 0.001
	PM_2.5_ (μg/m^3^)	4.95	1.04	1.02–1.07	0.003
Lag 2	O_3_ (ppb)	3.31	1.00	0.97–1.04	0.394
	CO (ppm)	4.85	0.96	0.93–1.00	0.073
	NO_2_ (ppb)	4.15	1.03	1.00–1.07	0.028
	SO_2_ (ppb)	1.30	0.95	0.91–0.99	0.030
	PM_10_ (μg/m^3^)	6.49	1.02	1.01–1.03	0.002
	PM_2.5_ (μg/m^3^)	2.09	1.04	1.02–1.05	0.014
Lag 3	O_3_ (ppb)	3.92	1.03	0.99–1.06	0.053
	CO (ppm)	5.60	1.07	0.99–1.08	0.488
	NO_2_ (ppb)	2.79	1.02	0.99–1.06	0.125
	SO_2_ (ppb)	8.21	0.94	0.91–0.99	0.016
	PM_10_ (μg/m^3^)	6.43	1.02	1.01–1.03	0.004
	PM_2.5_ (μg/m^3^)	2.41	1.05	0.99–1.10	0.051
Mean Lag 4–8	O_3_ (ppb)	3.33	1.03	0.99–1.06	0.066
	CO (ppm)	5.52	1.03	0.99–1.07	0.311
	NO_2_ (ppb)	2.54	1.02	0.99–1.06	0.116
	SO_2_ (ppb)	2.82	0.97	0.92–1.01	0.117
	PM_10_ (μg/m^3^)	6.47	1.00	0.99–1.01	0.089
	PM_2.5_ (μg/m^3^)	3.84	0.93	0.89–0.98	0.011

**Table 5:** Results of adjusted Generalized Additive Model and the effect of pollutants on respiratory deaths in women (for 1 unit increase in CO and 10 units increase in all other pollutants)

	Lag	df	RR	95% CI for RR	*P*-Value
Lag 0	O_3_ (ppb)	7.89	1.01	0.97–1.05	0.616
	CO (ppm)	4.62	0.98	0.94–1.03	0.438
	NO_2_ (ppb)	3.80	1.06	1.03–1.10	< 0.001
	SO_2_ (ppb)	5.27	0.94	0.89–0.99	0.019
	PM_10_ (μg/m^3^)	4.53	1.03	1.01–1.04	< 0.001
	PM_2.5_ (μg/m^3^)	1.83	1.04	0.98–1.10	0.355
Lag 1	O_3_ (ppb)	4.49	1.02	0.98–1.06	0.270
	CO (ppm)	5.39	1.03	0.99–1.08	0.476
	NO_2_ (ppb)	6.29	1.05	1.04–1.09	0.005
	SO_2_ (ppb)	4.33	0.95	0.91–1.00	0.056
	PM_10_ (μg/m^3^)	5.72	1.01	0.99–1.03	0.063
	PM_2.5_ (μg/m^3^)	7.23	1.05	1.01–1.09	< 0.001
Lag 2	O_3_ (ppb)	7.19	1.03	0.99–1.07	0.178
	CO (ppm)	5.76	1.04	0.99–1.09	0.694
	NO_2_ (ppb)	3.63	1.04	1.01–1.08	0.010
	SO_2_ (ppb)	5.27	0.94	0.89–0.99	0.029
	PM_10_ (μg/m^3^)	6.61	1.01	0.99–1.03	0.113
	PM_2.5_ (μg/m^3^)	2.53	1.03	1.01–1.05	0.003
Lag 3	O_3_ (ppb)	6.45	1.03	0.98–1.06	0.174
	CO (ppm)	4.91	1.04	0.99–1.09	0.421
	NO_2_ (ppb)	2.22	1.05	1.02–1.09	0.003
	SO_2_ (ppb)	1.36	0.96	0.92–1.01	0.132
	PM_10_ (μg/m^3^)	5.96	1.01	0.99–1.03	0.071
	PM_2.5_ (μg/m^3^)	2.41	1.03	0.98–1.07	0.067
Mean Lag 4–8	O_3_ (ppb)	3.31	1.01	0.96–1.04	0.801
	CO (ppm)	5.29	0.98	0.96–1.01	0.124
	NO_2_ (ppb)	4.88	1.05	1.01–1.08	0.011
	SO_2_ (ppb)	4.61	0.95	0.90–1.00	0.052
	PM_10_ (μg/m^3^)	6.93	1.00	0.98–1.02	0.589
	PM_2.5_ (μg/m^3^)	2.68	0.95	0.90–1.01	0.131

Tables 6, 7 and 8 show the exposure-response relation between air pollutants and respiratory deaths in age groups under 18, 18–60 and over 60 years. None of the air pollutants were related with respiratory deaths in people under 18 years old. But in the 18 to 60 and over 60 years old age group, NO_2_, O_3_, PM_10_ and PM_2.5_ showed significant relations. RRs can be seen in Tables 6, 7 and 8.

**Table 6:** Results of adjusted Generalized Additive Model, about the effect of air pollutants on respiratory deaths in peoples under 18 years old (for 1 unit increase in CO and 10 units increase in all other pollutants)

	Lag	df	RR	95% CI for RR	*P*- Value
Lag 0	O_3_ (ppb)	2.01	0.93	0.86–1.01	0.073
	CO (ppm)	1.54	0.94	0.86–1.02	0.134
	NO_2_ (ppb)	1	1.01	0.97–1.08	0.826
	SO_2_ (ppb)	3.24	0.97	0.86–1.05	0.286
	PM_10_ (μg/m^3^)	3.48	1.01	0.98–1.05	0.353
	PM_2.5_ (μg/m^3^)	6.71	0.99	0.88–1.16	0.981
Lag 1	O_3_ (ppb)	2.61	0.95	0.87–1.02	0.153
	CO (ppm)	1.79	0.92	0.84–1.00	0.060
	NO_2_ (ppb)	3.79	0.98	0.91–1.06	0.647
	SO_2_ (ppb)	4.71	0.95	0.85–1.05	0.281
	PM_10_ (μg/m^3^)	5.02	0.99	0.96–1.03	0.864
	PM_2.5_ (μg/m^3^)	2.46	1.00	0.88–1.13	0.95
Lag 2	O_3_ (ppb)	4.52	0.95	0.88–1.03	0.246
	CO (ppm)	3.84	0.89	0.82–0.98	0.017
	NO_2_ (ppb)	1	0.98	0.91–1.05	0.559
	SO_2_ (ppb)	4.5	1.01	0.91–1.11	0.918
	PM_10_ (μg/m^3^)	5.03	0.99	0.97–1.03	0.894
	PM_2.5_ (μg/m^3^)	1	1.02	0.91–1.15	0.688
Lag 3	O_3_ (ppb)	2.38	0.94	0.87–1.01	0.107
	CO (ppm)	1	0.87	0.80–0.95	0.002
	NO_2_ (ppb)	5.41	0.99	0.93–1.07	0.901
	SO_2_ (ppb)	8.25	0.99	0.89–1.09	0.899
	PM_10_ (μg/m^3^)	2.75	1.00	0.97–1.03	0.973
	PM_2.5_ (μg/m^3^)	6.15	1.03	0.91–1.16	0.647
Mean Lag 4–8	O_3_ (ppb)	3.28	0.93	0.86–1.01	0.078
	CO (ppm)	1	0.91	0.83–0.99	0.034
	NO_2_ (ppb)	1.33	1.04	0.96–1.11	0.344
	SO_2_ (ppb)	5.14	0.94	0.85–1.07	0.272
	PM_10_ (μg/m^3^)	2.7	1.00	0.97–1.03	0.961
	PM_2.5_ (μg/m^3^)	1	1.04	0.92–1.17	0.523

**Table 7:** Results of adjusted Generalized Additive Model and the effect of pollutants on respiratory deaths in peoples 18–60 years old (for 1 unit increase in CO and 10 units increase in all other pollutants)

	Lag	df	RR	95% CI for RR	*P*- Value
Lag 0	O_3_ (ppb)	7.57	1.07	0.99–1.09	0.128
	CO (ppm)	6.33	0.99	0.93–1.04	0.549
	NO_2_ (ppb)	1	1.05	1.00–1.09	0.034
	SO_2_ (ppb)	3.19	0.96	0.90–1.02	0.195
	PM_10_ (μg/m^3^)	7.47	1.02	1.00–1.04	< 0.001
	PM_2.5_ (μg/m^3^)	3.25	1.05	0.96–1.13	0.198
Lag 1	O_3_ (ppb)	5.57	1.04	0.99–1.08	0.117
	CO (ppm)	5.14	0.97	0.93–1.03	0.345
	NO_2_ (ppb)	7.07	1.05	1.01–1.10	0.026
	SO_2_ (ppb)	1	0.96	0.90–1.02	0.206
	PM_10_ (μg/m^3^)	7.08	1.03	1.01–1.05	0.001
	PM_2.5_ (μg/m^3^)	2.87	1.06	1.02–1.06	< 0.001
Lag 2	O_3_ (ppb)	5.28	1.06	1.01–1.11	0.014
	CO (ppm)	4.98	0.98	0.96–1.04	0.597
	NO_2_ (ppb)	4.14	1.06	1.01–1.10	0.011
	SO_2_ (ppb)	1.61	0.95	0.88–1.01	0.087
	PM_10_ (μg/m^3^)	6.84	1.03	1.01–1.05	0.003
	PM_2.5_ (μg/m^3^)	3.16	1.04	1.01–1.07	< 0.001
Lag 3	O_3_ (ppb)	6.96	1.07	1.02–1.12	0.004
	CO (ppm)	6.99	0.97	0.92–1.02	0.250
	NO_2_ (ppb)	2.2	1.04	0.99–1.10	0.084
	SO_2_ (ppb)	8.61	0.95	0.89–1.01	0.120
	PM_10_ (μg/m^3^)	7.47	1.02	0.95–1.09	0.156
	PM_2.5_ (μg/m^3^)	3.21	1.02	0.95–1.09	0.094
Mean Lag 4–8	O_3_ (ppb)	8.61	1.04	0.99–1.09	0.119
	CO (ppm)	4.49	0.97	0.93–1.03	0.433
	NO_2_ (ppb)	2.7	1.03	0.98–1.07	0.243
	SO_2_ (ppb)	1	0.95	0.89–1.02	0.137
	PM_10_ (μg/m^3^)	7.73	1.02	1.00–1.04	0.017
	PM_2.5_ (μg/m^3^)	4.26	1.00	0.97–1.04	0.341

**Table 8:** Results of adjusted Generalized Additive Model and the effect of pollutants on respiratory deaths in peoples over 60 years old (for 1 unit increase in CO and 10 units increase in all other pollutants)

	Lag	df	RR	95% CI for RR	*P*- Value
Lag 0	O_3_ (ppb)	8.18	1.04	1.00–1.07	0.024
	CO (ppm)	4.39	0.98	0.95–1.02	0.312
	NO_2_ (ppb)	3.38	1.05	1.02–1.08	0.001
	SO_2_ (ppb)	2.58	0.96	0.92–0.99	0.033
	PM_10_ (μg/m^3^)	7.29	1.03	1.02–1.04	< 0.001
	PM_2.5_ (μg/m^3^)	2.64	1.03	1.01–1.06	0.039
Lag 1	O_3_ (ppb)	3.48	1.04	1.00–1.07	0.027
	CO (ppm)	6.44	0.97	0.93–1.00	0.054
	NO_2_ (ppb)	4.67	1.04	1.01–1.07	0.01
	SO_2_ (ppb)	2.91	0.95	0.91–0.99	0.023
	PM_10_ (μg/m^3^)	5.77	1.02	1.01–1.03	0.005
	PM_2.5_ (μg/m^3^)	2.55	1.04	1.02–1.08	0.002
Lag 2	O_3_ (ppb)	4.02	1.03	1.00–1.06	0.041
	CO (ppm)	7	1.01	0.96–1.07	0.232
	NO_2_ (ppb)	3.86	1.04	1.01–1.07	0.006
	SO_2_ (ppb)	3.57	0.95	0.91–0.98	0.009
	PM_10_ (μg/m^3^)	6.24	1.01	1.00–1.03	0.038
	PM_2.5_ (μg/m^3^)	1.7	1.03	1.01–1.05	0.007
Lag 3	O_3_ (ppb)	4.78	1.03	0.99–1.06	0.065
	CO (ppm)	5.09	1.03	0.99–1.07	0.212
	NO_2_ (ppb)	2.89	1.04	1.01–1.07	0.003
	SO_2_ (ppb)	1.34	0.95	0.91–0.98	0.01
	PM_10_ (μg/m^3^)	5.81	1.01	1.00–1.03	0.026
	PM_2.5_ (μg/m^3^)	2.11	1.02	0.98–1.04	0.265
Mean Lag 4–8	O_3_ (ppb)	3.18	1.03	0.99–1.06	0.078
	CO (ppm)	5.05	1.03	0.99–1.07	0. 241
	NO_2_ (ppb)	4.98	1.05	1.02–1.08	0.006
	SO_2_ (ppb)	7.87	0.92	0.89–0.96	0.001
	PM_10_ (μg/m^3^)	7.83	1.01	1.00–1.03	0.035
	PM_2.5_ (μg/m^3^)	1.35	0.96	0.92–1.01	0.11

In men, women and in all age groups CO and SO_2_ showed no direct relation with respiratory disease. SO_2_ was not related to increase respiratory mortality, maybe because it did not exceed WHO thresholds except on 6 days in 10 years.

In this study, in some situations, SO_2_ showed an inverse relationship with respiratory death. More investigation about the probable harvesting effect of SO_2_ was done according to the methods suggested by previous studies [15]. The average count of deaths in the previous 14 days (two weeks) was divided by the average counts of deaths 3 days after exposure to SO_2_ and the ratio was 1.02 for all deaths, 1.03 for males and females. The RR for SO_2_ after adjusting for day of the week, season, meteorological variables and auto-correlation was 1.02 (95% CI 1.01–1.03) and this auto-correlation was significant (*p* < 0.001) and shows the presence of harvesting. Also, the RR for death in percentiles above the 95th percentile compared to the 5th percentile was 1.11 and shows that death is higher in higher concentrations of SO_2_ which is rational.

## Discussion

Air pollution, as one of the most important environmental health problems is a major threat to human health and leads to many adverse health effects, the most important of which is on the respiratory system. Studies have been carried out on its acute and chronic effects on the respiratory system [16]. The aim of this study was to investigate the relationship between respiratory mortality and air pollution.

From 2005 to 2014, the number of respiratory mortality in both men and women has gradually increased in Tehran. As the corresponding increase in Age-Standardized Rates was negligible, we can conclude that this increase was nearly entirely due to changes in population, such as increase in the urban population of Tehran due to immigration. However, this population increase is less likely to have affected our results. In time series regression studies, the denominator (the underlying population size) is not part of the dataset, and is not a concern because in these models, we are usually interested in modeling variation in outcome from day to day or week to week, and population size is unlikely to change meaningfully over these timescales, and can be safely omitted from the analysis. Also in this study, we assessed the short-term effect of exposure; and population change cannot have a significant effect on short-term results [17].

In this study, maximum daily concentration of ozone (O_3_) was 77.09 ppb and lower than WHO guidelines (Table 2). O_3_ also was related to overall respiratory deaths, male respiratory deaths and respiratory deaths in people 18 to 60 years and in people over 60 years old. RR for overall respiratory deaths was 1.03 (95% CI: 1.01–1.05) per 10 ppb increase in O_3_ in lag 1. This relation between O_3_ and respiratory deaths has also been seen in other studies. Monthly concentration of O_3_ was related to respiratory death in people over 60 years old in Shiraz (South West Iran) and was RR = 1.04, 95% CI: 1.00–1.09 per one ppb increase in O_3_ [9], and in Kerman (south east of Iran), there was a significant relation between the monthly concentration of O_3_ and respiratory deaths in men and was RR = 1.03, 95% CI: 1.01–1.04 per one ppm increase in O_3_ [16]. Shahi et al. showed that respiratory admissions to hospitals were related to O_3_ in lag 0 to 7 days in Tehran, the highest RR was seen in lag 1.01 (95% CI: 1.01–1.02) [18]. In this study, the mean concentration of O_3_ was 24.66 ± 9.12 and more than Shiraz (17.49 ± 16.28 ppb) [9] and Kerman (0.02 ± 0.01 ppm) [16]. In the period of this study, on only 6 days the concentration of SO_2_ passed WHO thresholds.

In this study, NO_2_ had a significant relationship with respiratory mortality in both sexes and in groups aged 18 and over. Other studies have also seen a relation between nitrogen dioxide and respiratory mortality [19–21]. The strongest RR for NO_2_ in this study for overall death was 1.04 (95% CI; 1.02–1.04) in lag 1. In César et al.’s study in Brazil, the RR for respiratory deaths per 10 μg/m^3^ increase in NOx was 1.04 (95% CI: 1.01–1.06) [19]. In Zhang et al. in Beijing, China the RR for respiratory death was 1.01 (95% CI: 1.00–1.01) per 10 μg/m3 increase of NO_2_ in lag 0–2 days [21]. In a meta-analysis in China, the pooled estimate of RR for respiratory deaths was 1.01 (95% CI: 1.01–1.02) per 10 μg/m^3^ increase in NO_2_ [22], which is close to our results.

In Tehran, the major source of NO_2_ production is the transportation system. Therefore, it is necessary to increase the use of public transport and decrease the use of fossil fuels to reduce this ambient air pollutant.

In this study, particulate matters (PM_10_ and PM_2.5_) showed a direct relation with respiratory deaths which were RR = 1.03, 95% CI: 1.02–1.04 and RR = 1.05, 95% CI: 1.01–1.09 respectively. Similar to this study, in studies done by Yang et al., in Beijing, China, there was a 1.7% (95% CI: 0.01–3.32) increase of daily respiratory deaths associated with an interquartile range (IQR) increase in PM_10_ concentrations [23] and in a study by Wang et al., in Seoul, Korea the RR for respiratory death was 1.0001 (95% CI: 1.0001–1.0002) per 1 μg/m^3^ increase in PM_10_ in lag 0 and 1 [14]. Another study by Lopez et al., in the Canary Islands showed that the Rate Ratio for respiratory mortality was RR = 1.04 (95% CI: 1.01–1.08) by 10 μg/m^3^ increase in PM_10_ and the maximum effect of exposure to PM_10_ on respiratory death was on the same day [4]. Studies from England [24], and Kerman, Iran [16] also showed a significant relation between monthly concentrations of PM_10_ and respiratory deaths. In a study from Isfahan, Iran there was a significant relation between PM_10_ and the number of children admitted to hospitals due to respiratory problems [25]. In Wang et al.’s, study in Seoul, Korea (71.23 μg/m^3^) [14], Yang et al., in Beijing, China (96.3 μg/m^3^) [23], Khanjani et al, in Kerman, Iran (125.69 μg/m^3^) [16] and Mansourian et al, in Isfahan, Iran (118.04 μg/m^3^) [25] the mean of concentration of PM_10_ were higher than this study (69.75 μg/m^3^), but in Lopez et al, in the Canary Islands (37.7 μg/m^3^) [4] and a meta-analysis by Hansel et al, in England (20.7 μg/m^3^) [24] the mean concentration of PM_10_ was lower than this study. Particulate matter are one of the most important pollutants that affect human health. Particulate matter compounds and sizes vary in different places and this can explain different study results.

In this study, Carbon monoxide showed no significant relation, and SO_2_ showed inverse relations with respiratory death, that was likely caused by the harvesting effect.

In some studies CO and SO_2_ have shown relations with respiratory deaths. For example, monthly CO concentrations were related with respiratory deaths in Shiraz [9] and CO was also related with respiratory hospital admission in Tehran [18].

SO_2_ in the Canary Islands [4], Kerman, Iran [16], Taiwan [26], and Tehran [20], was reported to be related with respiratory death. Other studies from Kerman reported a relation between hospital respiratory admission and SO_2_ [27].

The effects of different ambient air pollutants, has been reported differently in different parts of the world. This variability may be related to different study designs, various statistical models and including or not including different confounders.

In this study and other similar studies [4, 9, 16, 25, 26], some air pollutants had more adverse effects on the health outcomes in men than in women. In this study only ozone showed a different result based on gender and was more effective on respiratory deaths in men. The reason might be that men spend more time in outdoor environments. One study conducted in Tehran showed that the concentrations of ambient (outdoor) PM_10_, PM_2.5_ and PM_1_ were higher than indoors [28].

In this study, older people were more vulnerable to exposure to pollutants than younger people. None of the air pollutants were related to respiratory death in people under 18 years old. But in the over 18 year age groups; NO_2_, O_3_, PM_10_ and PM_2.5_ showed relations with respiratory death.

Mobile and stationary sources are both responsible for air pollution in Tehran. Motor vehicles are the main source for especially CO, NO_2_ and PM_10_ in Tehran. Tehran also has a lot of factories and is an industrial hub. According to Mazaheri et al., mobile sources of air pollution are more important than stationary sources in making and emitting NO_2_ and CO in Tehran [29]. Wang et al [30] showed that the mass ratio of nitrate to sulfate is a good indicator of the relative importance of mobile to stationary sources of nitrogen and sulfur in the atmosphere and it is reasonable to use nitrate as an indicator of transportation emission and sulfate as an indicator of stationary emission. The nitrate/sulfate ratio in Tehran, in 2014 ranged from 1.17 to 1.21 for PM_10_, 1.16 to 1.40 for PM_2.5_, and 1.23 to 1.45 for PM_1_. The results indicated that mobile sources were more important source of PM in Tehran [28]. The dismissal of old cars, using less fossil fuels for transportation and heating; and the transfer of factories out of the city are recommended in order to decrease air pollution in Tehran.

Another source of air pollution are the Middle Eastern dust storms which have also affected western and central Iran (including Tehran). Regional and international efforts to reduce particle matter in the Middle East are necessary.

Other studies about the effects of air pollutants indicate that air pollution has also caused deaths and hospital admissions due to heart disease and road accidents in Iran [31–36]. This evidence further shows the importance of controlling air pollution in this country.

Some limitations of this study were that the impact of other variables such as the concentrations of other pollutants like NO, lead and total hydrocarbons **(**THC), or wind direction was not investigated. We did not have data about influenza epidemics in Tehran and we did not adjust for this variable. Finally, given the ecological nature of this study, results cannot be easily inferred to an individual level.

## Conclusions

The results indicate that ambient Particle matter, Ozone and Nitrogen dioxide can be responsible for a significant part of respiratory mortalities in Tehran. Additional efforts to control and reduce air pollutant is necessary.
